# Exploring the Dimensionality of Ethnic Minority Adaptation in Britain: An Analysis across Ethnic and Generational Lines

**DOI:** 10.1177/0038038515609030

**Published:** 2015-11-24

**Authors:** Laurence Lessard-Phillips

**Affiliations:** University of Manchester, UK; University of Birmingham, UK

**Keywords:** adaptation, ethnicity, generations, migration

## Abstract

In this article I explore the dimensionality of the long-term experiences of the main ethnic minority groups (their adaptation) in Britain. Using recent British data, I apply factor analysis to uncover the underlying number of factors behind variables deemed to be representative of the adaptation experience within the literature. I then attempt to assess the groupings of adaptation present in the data, to see whether a typology of adaptation exists (i.e. whether adaptation in different dimensions can be concomitant with others). The analyses provide an empirical evidence base to reflect on: (1) the extent of group differences in the adaptation process, which may cut across ethnic and generational lines; and (2) whether the uncovered dimensions of adaptation match existing theoretical views and empirical evidence. Results suggest that adaptation should be regarded as a multi-dimensional phenomenon where clear typologies of adaptation based on specific trade-offs (mostly cultural) appear to exist.

## Introduction

Despite migration not being a recent phenomenon, Britain has experienced, in recent decades, an increase in migratory flows. This has partly been prompted by increases and changes in migration patterns, especially in the last decade, but also by changes in immigration and citizenship policies through the 20th and 21st centuries that have encouraged post-colonial migration, mainly the migration of individuals of different ethnic origins than that of the majority (mostly white) British population (Castles and Miller, 2009; Hansen, 2000; Jivraj, 2013).

The ethnic minority groups under study in this article (Indian, Pakistani, Bangladeshi, Caribbean and African)^1^ have varying migration histories. Among the more ‘established’ ethnic groups in Britain are from the Caribbean, whose first important wave of migration was in the 1950s, and from India, who started arriving in greater numbers in the 1960s. The 1970s and 1980s saw an increase in migratory flows from Pakistan and Bangladesh, often in the form of family reunification. Migrants from Africa (either economic migrants, post-colonial migrants or refugees) came in greater numbers in the 1990s (Cheung and Heath, 2007; Lessard-Phillips et al., 2014). Mostly as a result of these changes, the share of ethnic minorities in the country has increased from 2.9 per cent in 1951 to 14 per cent of the population in 2011 (Cheung and Heath, 2007; Jivraj, 2012).

Hence, there has been a steady change in the ethno-national origins of the migrants and an increase in the ethnic diversity of the country as these migrants settled in Britain and formed (or brought) a family of their own. This, over time, has had an impact on the ethnic, cultural and religious landscape of the country, prompting various policy and public debates. There has also been an increased interest in issues related to the inclusion of ethnic minorities including, but not limited to, targeted inclusion policies, the introduction of measures of ethnicity in data, and an increase in academic interest in their (and their descendants’) long-term settlement experiences across generations, here defined as their adaptation.

One issue that has arisen in academic circles is that of the way in which to qualify those experiences. In other words, important questions have arisen as to what adaptation comprises (i.e. whether there are specific dimensions to adaptation) and how potential dimensions of adaptation, if they exist, link together when we examine those adaptation experiences. Various sociological approaches have hypothesised about the dimensions of adaptation and their linkage but these have rarely been tested. In this article, I aim to explore empirically such dimensionality and linkage in Britain, focusing on adaptation outcomes of ethnic minorities, to see how their adaptation patterns appear to match existing theoretical knowledge and assumptions.

## Defining Adaptation

Within the migration literature, conventional approaches to the study of the effect of migration on receiving societies usually examine the tools, processes and long-term effects of entry and settlement experiences of migrants (Castles, 2010; Massey et al., 1999). The terminology used to identify the long-term effects is varied and often discipline- and context-specific, usually centred on concepts such as assimilation, ‘the decline, and at its endpoint the disappearance, of an ethnic/racial distinction and the cultural and social differences that express it’ (to use Alba and Nee’s (1997: 863) definition as an example); integration, ‘the process of becoming an accepted part of society’ (Penninx et al., 2004: 141); incorporation, a more neutral term used by US scholars that uses the concept of societal membership as an endpoint for immigrants (GCIR, 2012); and multiculturalism, a concept with high levels of popularity in the policy field and in political theory, which can be understood as a process by which ethnic and cultural minorities are recognised and accommodated, usually via the means of national policies (Bloemraad et al., 2008). All these definitions have many commonalities, such as the focus on migrants and their descendants becoming members of a given society, but assume various processes and outcomes for such membership, as well as different roles for the population and institutions of the receiving countries.

In this article, in order to steer away from any assumptions about definitions and linkages stemming from the definitions defined above (and the theoretical approaches outlined below), I use the term adaptation:

a dynamic process of adjustment to a new society, which involves both the migrants and their descendants and the people and institutions of the host country working through their differences, and whose outcomes can be either positive or negative and can span [many] dimensions. (Lessard-Phillips, 2009: 4)

This definition, which I consider more theoretically neutral for the study of the phenomenon, allows for potential dimensionality (if it exists); different rates of adaptation in the dimension(s); does not assume that the process is unidirectional and includes multiple actors (either individuals and groups with and without a migration background or institutions) involved in the process.

Despite the multi-actor focus of this definition, in this article I only examine adaptation outcomes and their dimensionality among ethnic minority groups, analysing them over generations. Examining the structure of their adaptation into British society is important to assess broader issues of ethnic minority inclusion, an ever important academic and policy goal. This does not imply that adaptation should only concern minorities; given the definition used here I see adaptation as a broader phenomenon that concerns British society as a whole and where all individuals, groups and institutions have an active role in the process, but here only focus on an aspect of it.

## Theoretical Background

This article’s theoretical underpinnings can be mostly found in sociology and political science, where many (mostly American) scholars have theorised about adaptation (although not always under the same moniker) and its underlying dimensions. These are briefly discussed below. For a more detailed discussion, see Bean et al. (2012).

First there is *classical assimilation theory*, which was popularised by the Chicago School in the 1920s and whose main (simplified) premise is that that migrants should, usually but not exclusively over the course of their lifetime, start to assimilate with the mainstream of a given receiving society and that any distinctiveness between the native and the migrant populations should, over time, become blurred (Gordon, 1964; Park and Burgess, 1921; Warner and Srole, 1945). Gordon’s (1964) seminal work on the topic highlights important stages of assimilation, which are all necessary to achieve for an immigrant to be considered assimilated. These are: acculturation (language, customs and norms; a necessary first step); structural assimilation (cliques, clubs and institutions); marital assimilation (inter-marriage); identification assimilation (identity); attitude reception assimilation (lack of prejudice); behaviour reception assimilation (lack of discrimination); and civic assimilation (Gordon, 1964: 71).

The *‘new’ assimilation theory*, which arose out of criticisms of classical assimilation from proponents of segmented assimilation (see below) states that assimilation into the mainstream is still possible and a likely outcome, but that the process might be unequal and take more time to achieve for some groups (especially based on their ethnicity). This is argued to stem from structural and societal circumstances. In this approach, assimilation does not necessarily involve the complete abandonment of an immigrants’ socio-cultural heritage for it to be successful (Alba and Nee, 2003; Alba et al., 2011; Waters et al., 2010). Despite the presence of stages and the idea that assimilation may not necessarily occur across one generation, the general assumption about dimensionality in this approach seems that it is a generally uni-dimensional phenomenon. Some scholars, however, argue this view is mostly a difference in interpretation of the theory in US and European circles, and that the framework does allow for multi-dimensionality, bi-directionality and various rates of assimilation in many segments (Morawska, 2008; Waters, 2013).

As mentioned, *segmented assimilation theory* arose out of some scholars’ dissatisfaction with how classical assimilation dealt with explaining the more differentiated assimilation paths of ‘new’ (i.e. post-1965) immigrants to the United States. This framework states that the interaction between various acculturation processes and modes of incorporation (the community, societal and institutional contexts at the time of immigration and settlement) will lead different immigrant groups to three main paths of assimilation. These are: assimilation into the mainstream; downward assimilation into the ‘underclass’; and a mixture of socio-economic assimilation while retaining aspects of the home culture (Portes and Rumbaut, 2001; Portes and Zhou, 1993; Portes et al., 2009). With regard to dimensionality, segmented assimilation theory suggests a bi-dimensional process where acculturation processes play an important differentiating role alongside other factors, mainly socio-economic.

Steering away from the assimilation perspective allows us to examine other approaches dealing with the dimensionality of migrant adaptation. *Multicultural approaches*, for example, assume that multiple cultures and ethnicities will be able to co-exist and evolve in order to form a new societal ideal in the receiving country (Bean et al., 2012). In fact, according to such approaches, adaptation processes include a dimension including social and cultural practices which may evolve independently of adaptation in other dimensions or even preclude (or allow) certain dimensions of adaptation to strive at the expense of others (Bloemraad et al., 2008; Kymlicka, 1995). This idea of a trade-off between different dimensions of adaptation is also found in the work of Maxwell (2012, 2013), who argues that a trade-off exists between cultural adaptation, on the one hand, and political and economic adaptation on the other, for certain immigrant communities. This is in many ways similar to segmented assimilation as well as the work of Berry (1992), who saw various adaptation outcomes vary along axes of acculturation strategies. Other authors have also claimed that spatial adaptation (i.e. segregation or diversity) can either co-exist or function independently with other forms of adaptation (e.g. Musterd, 2003; Phillips, 2007), with some arguing that area-level deprivation rather than ethnic segregation is key to understanding adaptation outcomes (Demireva and Heath, 2014; Kapoor, 2013).

The dimensionality argument has also been articulated by scholars studying refugees and refugee integration policy, where social and structural components are of importance (see Cheung and Phillimore, 2014 for details). For example, the model of refugee integration of Ager and Strang (2008: 170) implies that there are various domains to integration: markers and means (employment, housing, education and health); social connection (social bridges, social bonds and social links); facilitators (language and cultural knowledge and safety and stability); and foundation (rights and citizenship). Within this framework, while no domain is defined as more important, facilitators may act as important barriers to refugee integration.

This brief review of literature allows us to see that there is no clear consensus on the dimensionality of adaptation and the linkages between the different dimensions. Recent research has established that a certain dimensionality appears to exist, but that more investigation is required.

## Past Research

Empirical research tends to focus more on the individual analysis of various dimensions of adaptation rather than an assessment of the actual number of dimensions present in the inter-generational ‘migrant experience’. According to Bean and colleagues (2012), academic research on adaptation appears to be grouped under four main headers: socio-economic (including education, labour market outcomes); socio-cultural/linguistic (identity, religious affiliation and family formation practices); spatial (segregation); or political (electoral and non-electoral participation).

British research, which has generally steered away from an adaptation discourse, focusing more on the outcomes of ethnic minorities rather than migrants per se (Waters, 2013), is a prime example of research where analytical focus has mostly been on the adaptation of migrants and ethnic minorities in specific domains. Examples of such research in the quantitative realm includes analyses of adaptation into the socio-economic (Cheung and Heath, 2007; Dustmann and Theodoropoulos, 2010; Li, 2010; Platt, 2005; Rothon et al., 2009), socio-cultural (Bisin et al., 2008; Cinnirella and Hamilton, 2007; Dustmann and Fabbri, 2005; Manning and Roy, 2010; Muttarak and Heath, 2010), political (Anwar, 2001; Fieldhouse and Cutts, 2008; Heath et al., 2011) and spatial (Phillips, 2007) domains. In research focusing on the experience of adaptation, which has tended to be more qualitative in nature, research has provided in-depth understandings of the mechanisms underlying specific adaptation outcomes for particular groups (see, for example, Bhatti, 2011; Campbell and McLean, 2002; Dale et al., 2002; Hussain and Bagguley, 2005). This research highlights the importance of acknowledging the complexity and inter-relationships of adaptation outcomes, something rarely studied at the aggregate level.

While quantitative research allows exploring the determinants of adaptation in particular dimensions in more detail, it is weak on the extent to which these dimensions ‘bundle’ together. Exceptions come from Koopmans (2010) and Phillips (2007), who simultaneously explored the socio-economic and spatial adaptation outcomes of migrants and ethnic minorities, deemed to occur simultaneously, hence hinting towards the bundling of such aspects into a sole dimension. Battu and Zenou (2010) and Dustmann and Fabbri (2005), on the other hand, examine the separation of the cultural and socio-economic dimensions of adaptation, much as the one put forward by the segmented assimilation framework. Maxwell (2012) has recently argued for taking the multidimensionality of the integration experience into account, mostly focusing on a segmented assimilation-type trade-off between social integration (or exclusion) and economic and political integration. In their recent work on refugee integration, Cheung and Phillimore (2014) investigated the relationship between employment and social capital and pushed for further work on the connections between different domains of adaptation.

The dimensionality of adaptation has also been examined in other contexts, using an analytical method similar to that used in this article. Bean et al. (2012), for example, looked at the structure of second-generation incorporation in US and European cities and found that more inclusive places allowed for a greater dimensionality of outcomes, and hence more varied opportunities for advancement for the second generation. Williams and Ortega (1990) tested the structure of Gordon’s stages of assimilation and found that the stages of assimilation could be reduced to three dimensions: structural (economic); cultural; and receptional (attitudes).

The various assumptions of the theoretical frameworks outlined above as well as the relative lack of research looking simultaneously at the dimensionality of adaptation and their linkages opens the door for a global overview of the phenomenon in the British context. This is what I am attempting to do in this article. Analytically speaking, my main aim is to establish whether typical indicators of adaptation found in the literature do measure one latent concept (i.e. a one-dimensional version of adaptation), or whether we can speak of different dimensions of adaptation for ethnic minorities in Britain, using recent British data. I then examine whether specific groupings of adaptation exist among ethnic groups in British data, in order to establish which dimensions of adaptation, if any, go hand in hand when looking at individual outcomes. Finally, I investigate whether membership in these different groupings vary across ethnic groups, with a focus on generational differences, given the importance of these aspects in shaping adaptation, as highlighted by the theoretical frameworks above.

## Data

The data used to explore ethnic minority adaptation in the British context comes from the Ethnic Minority British Election Survey (EMBES; Heath et al., 2012). Conducted in 2010 (around the time of the General Election), EMBES is a nationally representative survey of the main ethnic minority groups in Britain: Indian; Pakistani; Bangladeshi; African; and Caribbean (Howat et al., 2011). The questions covered by EMBES focused mostly on the political behaviour of respondents, but also measured aspects important to the understanding of adaptation in other spheres. It is, all in all, one of the best sources to study ethnic minority adaptation. The study consisted of both face-to-face and self-completion questionnaires (with respective response rates of 66% and 35%; given the low response rate, questions from the self-completion questionnaire are not considered in these analyses). The total sample size of the survey is 2787 respondents (Howat et al., 2011). For the purposes of this article, which only includes valid responses on the selected variables, the analytical sample size is 1628.^2^ Weights controlling for selection probability and non-response are included in all analyses.

## Analyses

Three different types of analyses are being produced in order to examine the dimensionality of adaptation, the linkages between the dimensions (if more than one exist), as well as the differences in adaptation across ethnic and generational lines. The section below highlights the results stemming from these analyses.

### Dimensions of Adaptation

In the first instance, I performed factor analysis on selected indicators of adaptation to help uncover whether the chosen indicators measure the dimensions of adaptation most often found in the literature. Factor analysis allows measuring the relationships between variables, to see whether, taken together, they measure a single (or fewer) latent concepts (Acock, 2008). To analyse whether the chosen indicators measure one latent concept, a Principal Components Factor (PCF) approach is used.

The variables of interest for the factor analysis were selected according to the outlined theoretical and empirical knowledge above and include the following measures: education and occupation (typical measures of socio-economic adaptation); political engagement, feelings of influence in politics and voting at the General Election (typical indicators of political adaptation); socio-economic composition of the neighbourhood, ethnic density and diversity in the neighbourhood (typical measures of spatial adaptation); language, ethnicity of friends and spouse^3^ (typical measures of cultural adaptation); importance of British identity (typical measure of identity/cultural adaptation).^4^ More detail about the variables, their coding and their descriptive statistics can be found in Table 1. Using the approach suggested by Bean et al. (2012) to capture the dimensionality of adaptation, I coded the variables highlighted above in a way that higher codes indicate higher levels of adaptation, as suggested by the literature. This intrinsic coding hierarchy, necessary for conducting the analyses proposed here, allows for an estimation of adaptation that does not necessitate using the ‘majority’ population (however defined) as a comparative benchmark. Given that most of the selected indicators are measured by dichotomous or ordinal variables, the factor analysis performed uses a polychoric correlation matrix to deal with this fact (using the *polychoric* command in Stata; see Kolenikov and Ángeles, 2004 for more detail).

**Table 1.** Variables used in the analyses (N=1628).

Concept	Variable(s) used	Categories/numbers used	Distribution(mean, %)
Education	Imputation based on: British qualifications and foreign qualifications	GCSE & below A-level Below degree Degree	32.0% 18.3% 13.4% 36.4%
Class	Type of work	Never worked Semi-skilled or unskilled manual work Skilled manual work Foreman/ supervisor of other workers & Small business owner Sales or services Clerical Manager or senior administrator Professional or higher technical work	8.3% 13.2% 13.2% 4.8% 14.1% 8.9% 12.3% 25.3%
Non-electoral political participation	Additive scale; sum of: Protest/Petition/Boycott in last 12 months	0 (no non-electoral participation) 1 2 (two or more instances).	71.4% 18.8% 9.8%
Political influence	Influence on politics	Scale from 0 (no influence) to 10 (a great deal of influence)	2.7
Voting	Voted at 2010 Election	Did not vote Voted	26.5% 73.5%
IMD	Index of Multiple Deprivation, Percentiles	Percentiles (1- most deprived, 100- least deprived – 0/1 scale)	0.31
Percentage White	Derived from percentage non-white groups in Lower Layer Super Output Area (LSOA)	Proportion (0/1 variable)	0.63
Diversity index	Derived from percentage of ethnic groups in respondents’ LSOA.	Scale from 0 to1 based on calculated index.	0.92
Proportion of non-co-ethnics	Reversed proportion of co-ethnic in LSOA	Proportion (0/1 variable).	0.86
Language	English as main language	NoYes	33.8% 66.2%
Ethnicity of friends	Friends of same ethnicity/religion	All of them Most of them About half of them A few of them None of them	5.2% 43.5% 26.4% 22.7% 2.2%
Ethnicity of spouse	Spouse/partner’s ethnic group	Same No spouse White	53.5% 44.0% 2.5%
British identity	Most important identity: British or ethnicity	(Black/Asian) not British More (Black/Asian) than British Equally (Black/Asian) and British More British than (Black/Asian) British not (Black/Asian)	10.1% 20.8% 49.5% 16.7% 3.0%
Ethnicity	Ethnicity	Asian - Indian Asian - Pakistani Asian - Bangladeshi Black - CaribbeanBlack - African	40.3% 18.3% 7.4% 15.2% 18.9%
Generation	Derived from country of birth	Born outside UK Born in UK	59.6% 40.4%
Sex	Sex	Man Woman	51.4% 48.7%
Age	Age	Continuous	37.2

The results from the PCF analyses show that the selected variables seem to measure more than one latent component. In fact, we seem to get four distinct components of adaptation (see Tables 2 and A1 (online) for more detail). In the first component, which has the highest eigenvalue and proportion of variance explained, measures of deprivation, proportion of Whites in the area; and the level of diversity are strongly inter-related and appear to measure the spatial dimension of adaptation. In the second component, indicators of educational attainment, class and, to a certain extent, non-electoral participation can be seen as measuring a specific dimension based on socio-economic status. The third component is related to political identity, where indicators of political participation and British identity are related in measuring adaptation. This suggests that identity may operate independently from other aspects of cultural adaptation, which is the fourth and final component showing up in the analyses, where indicators of language, friendships and inter-marriage load together.^5^

**Table 2.** Characteristics of uncovered factors.

Factor	Eigenvalue	Main associated variables and factor loadings
(1) Spatial	2.83	IMD (0.56); % White (0.91); diversity (0.92); % non-co-ethnics (0.84)
(2) Socio-economic	1.66	Education (0.79); class (0.82); NE participation (0.42)
(3) Political identity	1.61	NE participation (0.47); feelings of influence (0.44); voting (0.71); Britishness (0.68)
(4) Cultural	1.51	Language (0.68); ethnicity of friends (0.63); ethnicity of spouse (0.69)

*Note*: IMD = Index of Multiple Deprivation; NE = non-electoral.

*Source*: EMBES. Rotated results (varimax rotation). PCF analysis. The factor loadings included are over 0.40. Full table of factor loadings in Table A1 (online).

**Figure 1.**
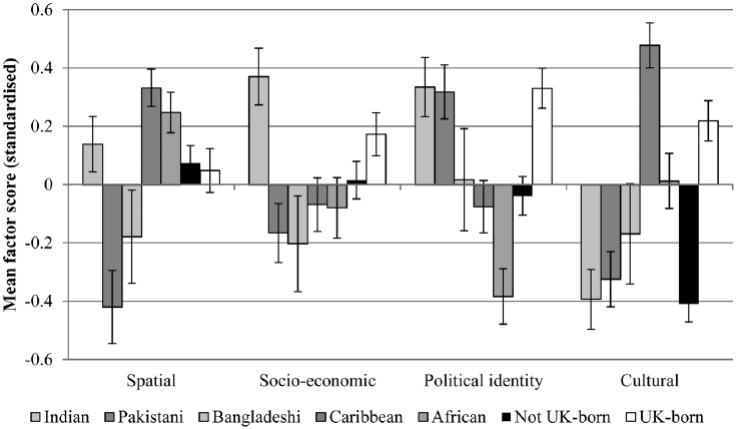
Mean standardised factor scores (PF) by ethnic group. *Source*: EMBES.

Given that the PCF analysis shows us that the indicators of adaptation measure more than one latent concept, I then generate four factor scores using a Principal Factor (PF) method, as PCF approaches are not suitable for generating more than one factor (Acock, 2008). This creates four distinct sets of numerical values related to the level of adaptation of each respondent in each dimension, with higher factor scores indicating higher levels of adaptation.^6^ Figure 1 shows the mean standardised factor scores in the different dimensions for the different ethnic groups and according to country of birth.

In terms of spatial adaptation, the results show that the Caribbean and African groups have higher mean factor scores in that dimension, followed by the Indian group, whereas the Pakistani and Bangladeshi groups, on the other hand, have the lowest scores. This generally reflects existing evidence (Phillips, 2007). With regard to the socio-economic dimension, the Indian group, consistent with research on ethnic inequalities in education and occupation (Li, 2010; Rothon et al., 2009), has higher mean factor scores than the other groups, especially the Pakistanis and Bangladeshis. Regarding political identity, the Indian and Pakistani groups have higher mean scores; Bangladeshi and Caribbean groups have average scores, whereas African respondents have the lowest mean score in that dimension. This is, again, consistent with existing research (Fieldhouse and Cutts, 2008; Heath et al., 2011). Finally, in terms of the cultural dimension, the Indian, Pakistani and Bangladeshi groups all have low mean scores, followed by the Africans, with the Caribbean group having the highest mean score, fitting results highlighted by Maxwell (2012). Results comparing respondents according to country of birth indicate that being UK-born is associated with higher adaptation scores in the socio-economic, political identity and cultural dimensions, but that non-UK born respondents score higher in terms of spatial adaptation and much lower in terms of cultural adaptation.

The results presented in this section are much in line with the idea that adaptation is a multi-dimensional concept, with important between-group variations, which are consistent with existing evidence. The next step is now to explore whether the factors of adaptation outlined above function independently or whether individuals cluster into groupings, where adaptations into specific dimensions co-exist.

### Linkages between Dimensions

Having established that more than one factor is measured by the variables chosen and having reduced these variables to four latent factors of adaptation, I perform a cluster analysis on the standardised factor scores, using complete linkage (i.e. clustering among the most dissimilar cases). Whereas factor analysis looks at inter-relationships between variables, cluster analysis is an exploratory method that helps in determining how cases group together based on their responses to specific variables (Everitt et al., 2011). This allows seeing whether individuals cluster into these specific, separate, dimensions of adaptation, or whether they tend to cluster into more complex groupings of adaptation. In other words, I am trying to see whether having a higher score in one dimension precludes high scores in other dimensions, or whether there exist groupings where high/low scores in more than one dimension of adaptation are emphasised. The latter would suggest a more complex adaptation process than suggested by the factor analysis and more in line with some of the theoretical explanations outlined above.

Results from the cluster analysis of the four factor scores (presented in Table 3) suggest that there are four main groupings of adaptation that follow a more complex structure than one focusing on adaptation into specific dimensions. The decision to use four groupings was derived from using information from the Duda-Hart index – the largest value in combination with the lowest pseudo-T-square value (Duda et al., 2001) – for helping in choosing the number of relevant clusters.

**Table 3.** Clusters of adaptation.

	Factors of adaptation (standardised mean score)			
	Spatial	Socio-economic	Political identity	Cultural
(1) Cultural and political exclusion (393)	0.35	−0.08	−0.40	−1.06
(2) Overall adaptation (718)	0.43	0.47	0.48	0.22
(3) Politically and economically disenfranchised, cultural inclusion (265)	0.14	−0.64	−0.52	0.76
(4) Isolated and engaged (252)	−1.65	−0.19	0.48	−0.42

*Source*: EMBES.

The four main groupings of adaptation are as follows. The first cluster comprises individuals with generally high levels of spatial adaptation, close to average levels of socio-economic adaptation, low levels of political identity and very low levels of cultural adaptation. Such individuals are thus grouped in a cluster entitled the ‘Cultural and political exclusion’. The second cluster, the most numerous one (comprising about 44% of the sample), includes individuals with overall high levels of adaptation in all dimensions. They are in the ‘Overall adaptation’ group. The third cluster comprises individuals with high levels of cultural adaptation, coupled with low levels of economic and political identity adaptation. These respondents appear to be excluded from participation in political and economic life. I call them the ‘Economically and politically disenfranchised with cultural inclusion’ group. Finally, a fourth cluster comprises a small proportion of respondents that exhibit very low levels of spatial adaptation and low levels of cultural adaptation but high levels of political identity; they are part of the ‘Isolated but politically engaged’ cluster. More than half of this cluster is comprised of respondents from the Pakistani group.

There thus appear to be specific groupings of adaptation outcomes among the EMBES respondents, which suggest that specific typologies of ethnic minority adaptation involving specific trade-offs, especially along cultural and political lines, and various levels of exclusion may well exist. The next step involved is thus to examine which groups are more likely to fall within those categories.

## Ethnic Effects and Differences across Generations

**Figure 2.**
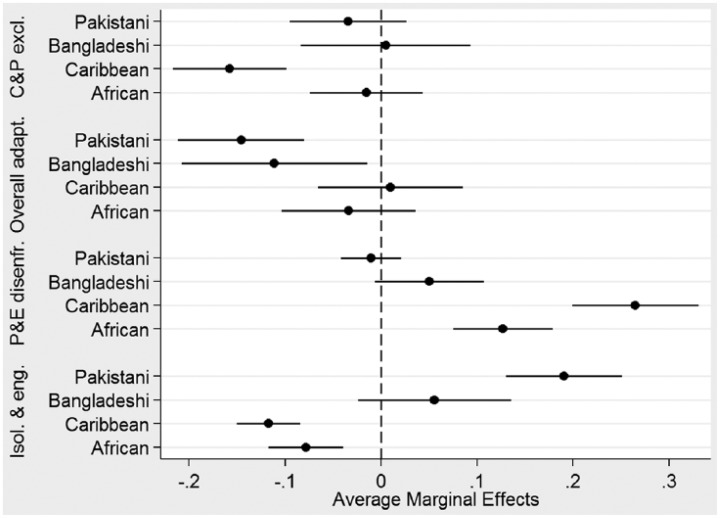
Average marginal effects of ethnicity on membership in adaptation clusters (Indian group as reference). *Source*: EMBES.

**Figure 3.**
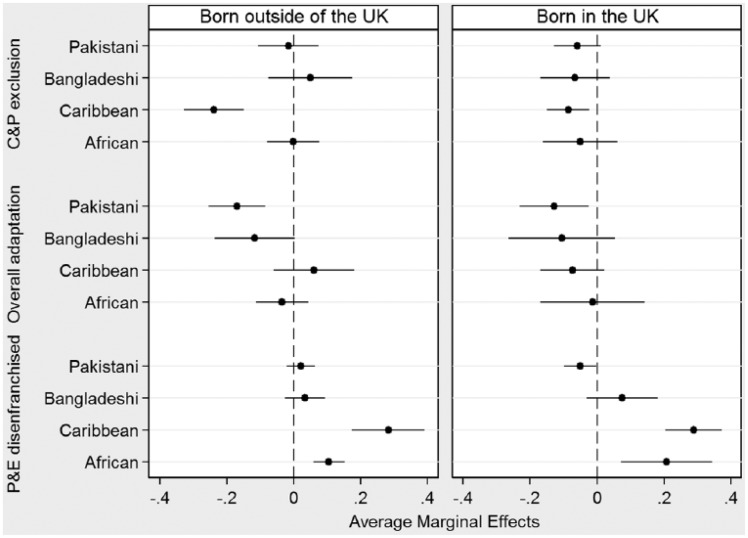
Average marginal effects of ethnicity on membership in adaptation clusters, by country of birth (Indian group as reference). *Source*: EMBES.

Given the unordered categorical nature of the dependent variable (i.e. the clusters from Table 3), multinomial logistic regression is used to indicate the probability of being in a specific adaptation grouping for the variables included in the model (Agresti, 1996). In these analyses, ethnicity and country of birth are included as independent variables and age and gender as controls. In order to compare effectively ethnic and generational effect sizes, average marginal effects (AMEs) are presented here; AMEs represent the average effect of the independent variables on being in specific categories of the dependent variables (see Mood, 2010 for a discussion on the use of marginal effects in logistic regression). In the analyses, I use the Indian group as the reference ethnic group in the regressions, as it is often found to exhibit high levels of adaptation in the socio-economic domain (see Cheung and Heath, 2007). The results, which compare the ethnic effects in relation to the reference category and hence allow exploring adaptation outcomes across the different ethnic groups, are highlighted in Figures 2 and 3.^7^ A coefficient to the left of the dotted line indicates a negative effect on being in a specific cluster compared to the reference group; one to the right, a positive effect. The figures show important group variations in adaptation cluster membership.

With regard to overall ethnic effects, portrayed in Figure 2, I find that, in comparison to the Indian group, Caribbean respondents are less often found in the ‘cultural and political exclusion’ grouping whereas Pakistani and Bangladeshi respondents are less often found in the ‘overall adaptation’ grouping. In relation to the Indian group, it is respondents from the Caribbean and African groups that tend to be most often found in the ‘politically and economically disenfranchised’ group, but less often in the ‘isolated and engaged’ group. With regard to that last grouping, and as expected given the composition of that group, it is respondents of Pakistani origins that are most often found there.

In terms of the effect of the other variables in the model (shown in the online appendix in Table A2), I find that there are no significant gender effects (aside from a small negative effect (*p* < .10) of being female for membership in the ‘overall adaptation’ group). There is a small positive effect of being older for membership in the ‘overall adaptation’ group and a small negative effect for being in the ‘politically and economically disenfranchised’ group. Being born in the UK is negatively related to membership in the ‘culturally and politically excluded’ cluster, whereas it is positively related in the ‘overall adaptation’ cluster. Given this, I explore ethnic differences in adaptation cluster membership by country of birth in Figure 3.

Turning to these ‘generational’ differences, which exclude the fourth cluster because of small numbers, the differences in the patterns of clustering do not necessarily depart from the ethnic effects outlined above, especially for respondents born outside of the UK. Regardless of nativity, and compared to their Indian peers, Pakistani respondents are less often found in the overall adaptation cluster; Caribbean respondents are less often in the ‘cultural and political exclusion’ cluster (with reduced effects in the UK-born group); and Caribbean and African respondents are most often found in the ‘politically and economically disenfranchised’ cluster. The gender and age effects are similar to the ones outlined earlier.

These results are indicative of the fact that while the multiple dimensions of adaptation cluster in specific ways, this clustering also seems to be quite group-specific, especially in terms of ethnicity. They also seem to fit consistent theoretical and empirical patterns that emphasise the varying importance of acculturation processes and civic inclusion across groups given the importance of the cultural and political dimensions in driving the structure of adaptation outcomes.

## Conclusion

In this article, my aim was to explore the structure of ethnic minority adaptation in Britain in an aggregate manner by examining whether it is of a multi-dimensional nature and, if so, how the uncovered dimensions group together for individuals with a (close or distant) migration background. The results outlined above appear to suggest that adaptation, at least in terms of ethnic minority outcomes, is of a multi-dimensional nature. Not only does there seem to be distinct dimensions of adaptation (spatial, socio-economic, political identity and cultural), but there also seems to be a clear pattern of adaptation groupings. In fact, what we encounter are four distinct adaptation typologies: (1) a main one where individuals do quite well with regard to adaptation in all dimensions; (2) a second where individuals are culturally and politically isolated; (3) a third featuring low levels of socio-economic and political adaptation but high levels of cultural adaptation; and (4) a fourth grouping comprising of spatially, socio-economically and culturally segregated individuals with high levels of political adaptation.

On the one hand, the results suggest that theoretical emphasis on acculturation and cultural trade-offs may well exist for those not exhibiting high levels of adaptation. Positive cultural adaptation, at least in the EMBES sample, appears to come at the price of lack of adaptation in the socio-economic and political dimensions. More needs to done to understand what types of mechanisms come into play here. On the other hand, the results also highlight the importance of (lack of) spatial adaptation, lending even more credence to the argument that segregation, not only defined in terms of ethnic segregation, is an important component of adaptation. This is definitely a factor worth examining in more detail, especially in its relationship with ‘typical’ factors of economic or political adaptation, for example.

Adaptation thus appears to be of a multi-dimensional nature; but is such dimensionality simply a sign of a ‘lagged’ level of adaptation that seems to disappear across generations? To a certain extent. Analyses of differences by country of birth in the dimensionality of adaptation show improvements in adaptation scores for the UK-born groups, which would fit an idea of ‘generational advancement’. Yet some of the ethnic patterns in the clustering of adaptation remain for UK-born groups; especially for the Pakistani, Caribbean and African groups. This persistence of ethnic (dis)advantages is well documented but nevertheless represents an issue to be tackled, especially with regard to identifying sources of such lag, where socio-economic status, as well as exclusion and discrimination in all domains could play a role in determining the position of certain groups in the various adaptation groupings.

Of course, the way in which the domains of adaptation are defined, conceptualised and measured plays a big role on the results and conclusions above and should not be ignored. If anything, the results from this article call for a deeper examination of the definition, conceptualisation and measurement of adaptation, especially in the British context, something that is being increasingly acknowledged. Health, relatively absent from the theoretical frameworks outlined above, but emphasised by the Ager and Strang (2008) framework of refugee integration, should also be explored. Further research including more ethnic groups (and generations) would be required in order to provide more granularity to the results. Moreover, taking into account ethnic group heterogeneity would also be required, which existing measurements of ethnicity tend to ignore (Burton et al., 2010), as well as measuring adaptation outcomes for society as a whole (i.e. including the ‘majority’ population) to see whether adaptation outcomes are similar for British society as a whole. Whether generational differences reflect changes in behaviour or changes in outcomes also remains to be investigated, as is the potential fuzziness of the clustering within the dimensions and issues of causality, ideally with the help of longitudinal and qualitative data. These are essential and important undertakings that will allow for an even better understanding of ethnic minority adaptation in Britain, which has been demonstrated to be a multi-dimensional and complex phenomenon.

## Supplementary Material

Supplementary material

## Notes

1. These are the main ethnic groups present in the Ethnic Minority British Election Study, the data used in the article. They are also the most numerous ethnic groups in the 2011 Census: Indian (2.5%); Pakistani (2%); Bangladeshi (0.8%); Caribbean (1.1%) and African (1.8%) (Office for National Statistics, 2012).

2. The proportion of missing values is usually < 10 per cent per indicator. The exception is the variable for educational attainment, where the number of unclassifiable qualifications (i.e. other qualifications) is quite high.

3. Note that inter-marriage can be seen both as a measure and outcome of adaptation; here I treat it as a measure, following Gordon’s framework.

4. Given the scope of the article, I have excluded perceptions of discrimination because of the fact that the act of discrimination, which hinders adaptation, is linked to the attitudes of the ‘majority’ population but here is measured at a subjective level. Analyses including discrimination show, however, that it may be negatively linked to cultural adaptation and considered a separate dimension in itself, thus matching Gordon’s framework.

5. All indicators load onto one component when analysed separately.

6. Do note that the structure of the factors is similar in the PCF and PF solutions.

7. Graphs produced using Jann’s (2014) *coefplot* command. Tables with coefficients and AMEs can be found in the online Appendix (A2–A4).
