# Supplementary material for: Exploring the Dimensionality of Ethnic Minority Adaptation in Britain: An Analysis across Ethnic and Generational Lines
Source: Sociology. 2015 Nov 24;51(3):626–45. doi: 10.1177/0038038515609030 (PMC5424857; doi:10.1177/0038038515609030)
Supplement: Supplementary material [file Lessard-Phillips_online_appendix_V3.pdf]

Online appendix

Table A1 Full factor analysis results

	(1) Spatial	(2) Economic	(3) Political identity	(4) Cultural	Uniqueness
Eigenvalue	2.83	1.66	1.61	1.51	
Proportion of variance	0.22	0.13	0.12	0.12	
Education	0.15	**0.79**	-0.10	0.02	0.34
Class	0.10	**0.82**	0.06	0.02	0.32
Non-electoral participation	-0.01	**0.42**	**0.47**	0.12	0.60
Political influence	0.05	-0.23	**0.44**	0.03	0.75
Voting	-0.13	0.03	**0.71**	-0.19	0.44
Index of Multiple Deprivation	**0.56**	0.28	0.20	-0.21	0.52
% White	**0.91**	0.08	0.07	0.02	0.16
Herfindahl index	**0.92**	0.06	-0.08	0.06	0.13
% of non-co-ethnics	**0.84**	0.05	-0.18	0.16	0.23
Language	0.11	0.19	0.21	**0.68**	0.44
Ethnicity of friends	0.21	0.00	0.12	**0.63**	0.55
Ethnicity of spouse	0.04	-0.10	-0.27	**0.69**	0.43
British identity	-0.07	-0.07	**0.68**	0.23	0.47

Source: EMBES. Rotated results (varimax). PCF analysis. Factor scores over 0.4 in bold.

Table A2 Multinomial regression results (coefficients and AMEs), all respondents (N=1,628)

Pseudo R^2: 0.12<br>Log likelihood: -1865.09	Cultural & political exclusion		Overall adaptation (ref)	Politically & economically disenfranchised		Isolated & engaged	
	Coefficient (SE)	AME (SE)	AME (SE)	Coefficient (SE)	AME (SE)	Coefficient (SE)	AME (SE)
Ethnic group (ref: Indian)							
Pakistani	0.24	-0.03	-0.15 ***	0.17	-0.01	1.21 ***	0.19 ***
	(0.18)	(0.03)	(0.03)	(0.30)	(0.02)	(0.19)	(0.03)
Bangladeshi	0.28	0.00	-0.11 *	0.83 *	0.05	0.59*	0.06
	(0.25)	(0.04)	(0.05)	(0.34)	(0.03)	(0.29)	(0.04)
Caribbean	-0.82 ***	-0.16 ***	0.01	1.65 ***	0.27 ***	-1.77 ***	-0.12 ***
	(0.23)	(0.03)	(0.04)	(0.23)	(0.03)	(0.42)	(0.02)
African	0.01	-0.02	-0.03	1.17 ***	0.13 ***	-0.73 **	-0.08 ***
	(0.17)	(0.03)	(0.04)	(0.24)	(0.03)	(0.28)	(0.02)
Age	-0.01	-0.00	0.00 **	-0.04 ***	-0.00 ***	0.00	0.00
	(0.01)	(0.00)	(0.00)	(0.01)	(0.00)	(0.01)	(0.00)
Female	0.22	0.03	-0.04	0.18	0.01	0.07	-0.00
	(0.13)	(0.02)	(0.02)	(0.16)	(0.02)	(0.16)	(0.02)
Born in UK	-1.25 ***	-0.22 ***	0.17 ***	-0.13	0.03	-0.26	0.02
	(0.16)	(0.03)	(0.03)	(0.19)	(0.02)	(0.18)	(0.02)
Constant	0.16			-0.65		-1.24 ***	
	(0.25)			(0.35)		(0.33)	

Source: EMBES. ***p<0.001 **p<0.01, *p<0.05. Note that there are no coefficients generated for the reference category in the regression.

Table A3 Multinomial regression results (coefficients and AMEs), respondents born outside of the UK (N=946)

Pseudo R^2: 0.11<br>Log likelihood: -1110.40	Cultural & political exclusion		Overall adaptation (ref)	Politically & economically disenfranchised	
	Coefficient (SE)	AME (SE)	AME (SE)	Coefficient (SE)	AME (SE)
Ethnic group (ref: Indian)					
Pakistani	0.45	-0.02	-0.17 ***	0.91 *	0.02
	(0.23)	(0.05)	(0.04)	(0.45)	(0.02)
Bangladeshi	0.44	0.05	-0.12	0.91	0.03
	(0.30)	(0.06)	(0.06)	(0.53)	(0.03)
Caribbean	-1.10 **	-0.24 ***	0.06	1.95 ***	0.28 ***
	(0.35)	(0.05)	(0.06)	(0.39)	(0.06)
African	0.08	-0.00	-0.03	1.36 ***	0.10 ***
	(0.18)	(0.04)	(0.04)	(0.33)	(0.02)
Age	-0.00	-0.00	0.00	-0.03 **	-0.00 **
	(0.01)	(0.00)	(0.00)	(0.01)	(0.00)
Female	0.32 *	0.04	-0.08 **	0.53 *	0.03
	(0.15)	(0.03)	(0.03)	(0.24)	(0.02)
Constant	-0.09			-1.59 ***	
	(0.28)			(0.47)	

Source: EMBES. ***p< 0.001 **p< 0.01, *p< 0.05. Note that there are no coefficients generated for the reference category in the regression.

Table A4 Multinomial regression results (coefficients and AMEs), respondents born in the UK (N=682)

Pseudo R^2: 0.10<br>Log likelihood: -734.69	Cultural & political exclusion		Overall adaptation (ref)	Politically & economically disenfranchised	
	Coefficient (SE)	AME (SE)	AME (SE)	Coefficient (SE)	AME (SE)
Ethnic group (ref: Indian)					
Pakistani	-0.16	-0.06	-0.13 *	-0.48	-0.05 *
	(0.32)	(0.04)	(0.05)	(0.43)	(0.02)
Bangladeshi	-0.25	-0.07	-0.11	0.77	0.08
	(0.52)	(0.05)	(0.08)	(0.45)	(0.05)
Caribbean	-0.48	-0.09 **	-0.07	1.59 ***	0.29 ***
	(0.32)	(0.03)	(0.05)	(0.28)	(0.04)
African	-0.28	-0.05	-0.01	1.22 **	0.21 **
	(0.50)	(0.06)	(0.08)	(0.41)	(0.07)
Age	-0.02	-0.00	0.01 **	-0.07 ***	-0.01 ***
	(0.01)	(0.00)	(0.00)	(0.01)	(0.00)
Female	0.18	0.03	0.01	-0.04	-0.01
	(0.23)	(0.03)	(0.04)	(0.23)	(0.03)
Constant	-0.64			0.37	
	(0.46)			(0.44)	

Source: EMBES. ***p<0.001 **p<0.01, *p<0.05. Note that there are no coefficients generated for the reference category in the regression.
